# Synthesis and Characterization of Some New Tetraaldehyde and Tetraketone Derivatives and X-ray Structure of 1,1′-(4,4′-(2-(1,3-bis(4-Acetylphenoxy)propan-2-ylidene)propane-1,3-di-yl)bis(oxy)bis(4,1-phenylene))diethanone

**DOI:** 10.3390/ijms9061000

**Published:** 2008-06-13

**Authors:** Mustafa Er, Reşat Ustabaş, Ufuk Çoruh, Kemal Sancak, Ezequiel Vázquez-López

**Affiliations:** 1 Department of Chemistry Faculty of Arts and Sciences Karadeniz Teknik University, 61080-Trabzon, Turkey; E-mails: muser@ktu.edu.tr; ksancak@ktu.edu.tr; 2 Department of Middle Education, Educational Faculty, Ondokuz Mayis University 55200-Atakum-Samsun, Turkey; 3 Department of Computer Education and Instructional Technology, Educational Faculty, Ondokuz Mayis University 55200-Atakum-Samsun, Turkey; E-mail: ucoruh@omu.edu.tr; 4 Departamento de Química Inorgánica, Facultade de Ciencias-Química, Universidade de Vigo, 36200-Vigo, Galicia, Spain; E-mail: ezequiel@uvigo.es

**Keywords:** 1,3-bis(4-Acetylphenoxy)propan-2-ylidenepropane, 1,3-di-ylbis(oxy)bis(4,1-phenylene))diethanone, crystal structure, x-ray

## Abstract

Tetraketone and tetraaldehyde derivatives **2a–d** were synthesized via the reaction of ethene-1,1,2,2,-tetra-yl-tetramethylene tetrabromide (**1**) with hydroxyketone and aldehyde derivatives. The molecular structures were identifed by IR, ^1^H-NMR, ^13^C-NMR and MS analysis. The crystal structure of the title compound **2a**, C_38_H_36_O_8_, is reported. Its crystal data are: monoclinic, space group P 2(1)/n with cell dimensions of a=9.0395(12) Å, b=12.6114(17) Å, c=13.8166(18) Å, β=95.875(3), V=1566.8(4) Å^3^, F.W.=620.67, ρ_calc_=1.316 gcm^3^ for Z=2, μ=0.092 mm^−1^

## Introduction

Acetophenone (AP, phenylmethylketone or hypnone) is used in consumer fragrances and as an industrial solvent [1]. Acetophenone and its derivatives are important compounds for obtaining biologically active compounds. In general, acetophenones are an important constituent of effective therapeutics against mycobacteria [2]. Acetophenones are used to obtain benzofuran, and its ketoxime derivatives show antifungal activities [3]. In adddition, acetophenones which contain halogens are used to synthesize disubstituted 1,3-thiazole compounds that have selective human adenosine A3 receptor antagonist [4] as well as antifungal and antibacterial activities. Some acetophenone derivatives have antimicrobial activity against gram-positive bacteria and fungi [5] while others are used as herbicides [6]. Certain acetophenones carrying a hydroxyl group at C-2 have antimutagenic activity in *Salmonella typhimurium* [7]. Many acetophenones are found as natural products in plants [8] and fungi [9]. The oral administration of Paeonol (2-hydroxy-4-methoxy acetophenone) to rats is followed by rapid excretition in the urine as its sulphated derivative [10]. Some acetophenone semicarbazone and acetophenone oxime derivatives are used to obtain biologically active industrial polimers [11]. *o*-Hydroxyacetophenone oxime is an important analytical reagent for the gravimetric and colorimetric estimation of transition metals [12]. Acetophenone derivatives are very interesting model compounds as foreign substrates for biotransformation, because an enantiomer may be formed, which can be determined easily. These compounds have been effectively used as a building blocks for the asymmetric synthesis of drugs [13].

In a part of our study, we aimed to enhance the selectivity of these macromolecular compounds and the stability of the Schiff base formed with both various amines and heterocyclic moiety. So, we prepared macromolecular compounds having aldehyde and ketone functions connection by flexible bridge. In this study, tetraaldehyde and tetraketone derivatives **2a–d** were obtained from the reaction with ethene-1,1,2,2-tetra-yl-tetra methylene tetra bromide (**1**) with hydroxyaldehydes and hydroxyketones.

## 2. Experimental

### 2.1. Materials

Melting points were determined on a Gallenkamp melting point apparatus and are uncorrected. ^1^H-NMR and ^13^C-NMR spectra were recorded on a Varian-Mercury 200 MHz spectrometer. The IR spectra were measured as potassium bromide pellets using a Perkin-Elmer 1600 series FTIR spectrometer. The MS spectra were determined on a Micromass Quatro LC/ULTIMA LC-MS spectrometer. Elemental analyses was carried out on a C,H,N-O rapid elemental analyzer Hewlett-Packard 185 for C, H and N and results are with in 0.4 % of the theoretical values. All the chemicals were obtained from Fluka Chemie AG Buchs (Switzerland). Compound **1** was synthesized using the published methods [14].

### 2.2. Synthesis of 1,1′-(4,4′-(2-(1,3-bis(4-acetylphenoxy)propan-2-ylidene)propane-1,3-di-yl)bis(oxy)bis (4,1-phenylene))diethanone (***2a***)

4-Hydroxyacetophenone (0.04 mol) and potassium hydroxide (0.04 mol) were refluxed in absolute ethanol for 2 hours. 1,1,2,2-Tetra-yl-tetramethylene tetrabromide (0.01) was added to the reaction mixture which was refluxed for 20 hours. The mixture was filtered and the solvent was evaporated. The solid residue was recrystallized from chloroform-acetone (1:1) to give compound **2a** (yield 73 %; m.p. 440–441°K). Analysis (% Calculated/found) for C_38_H_36_O_8_ (Mw 620.7) C: 73.53/73.44, H: 5.85/5.82; IR (KBr) (ν, cm^−1^), 3045 (Ar-CH), 2938 (-CH), 1674 (C=O); ^1^H-NMR (DMSO-d_6_) δ (ppm) 2.51 (s, 12H, CH_3_), 4.99 (s, 8H, O-CH_2_), 7.04–7.92 (dd, 16H, J= 8.0 Hz, AA′XX′, Ar-H); ^13^C-NMR (DMSO-d_6_) δ (ppm) 26.35 (CH_3_), 64.90 (O-CH_2_), Ar-C: [114.50 (CH), 130.07 (C), 100.29 (CH), 161.99 (C)], 135.05 (C=C), 196.24 (C=O); MS: m/z 621.34 (M+1)^+1^.

### 2.3. Synthesis of (4,4′-(2-(1,3-bis(4-benzoylphenoxy)propan-2-ylidene)propane-1,3-diyl)bis(oxy)bis (4,1-phenylene))bis(phenylmethanone) (***2b***)

4-Hydroxybenzophenone (0.04 mol) and potassium hydroxide (0.04 mol) refluxed in absolute ethanol for 2 hours. 1,1,2,2-Tetra-yl-tetramethylene tetrabromide (0.01) was added to the reaction mixture, which was then refluxed for 20 hours. The mixture was filtered and the solvent was evaporated. The solid residue was recrystallized from DMF-ethyl alcohol (2:1) to give compound **2b** (yield 64 %; m.p. 467–468°K). Analysis (% Calculated/found) for C_58_H_44_O_8_ (Mw 869.0) C: 80.17/80.26, H: 5.10/5.14; IR (KBr) (ν, cm^−1^), 3065 (Ar-CH), 2970 (-CH), 1652 (C=O); ^1^H-NMR (DMSO-d_6_) δ (ppm) 5.07 (s, 8H, O-CH_2_), 7.13–7.57 (dd, 16H, J= 8.2 Hz, AA′XX′, Ar-H), 7.62–7.65 (m, 8H, Ar-H), 7.69–7.71 (m, 8H, Ar-H), 7.75 (m, 4H, Ar-H); ^13^C-NMR (DMSO-d_6_) δ (ppm) 64.96 (O-CH_2_), Ar-C: [114.57 (CH), 128.34 (CH), 129.17 (CH), 129.58 (C), 131.98 (CH), 132.06 (CH), 137.55 (C), 161.87 (C)], 135.12 (C=C), 194.30 (C=O); MS: m/z 869.31 (M+1)^+1^.

### 2.4. Synthesis of 2,2′-(2-(1,3-bis(2,4-dichloro-6-formylphenoxy)propan-2-ylidene)propane-1,3-diyl)bis (oxy)bis(3,5-dichlorobenzaldehyde) (***2c***)

3,5-Dichlorosalicylaldehyde (0.04 mol) and potassium hydroxide (0.04 mol) were refluxed in absolute ethanol for 2 hours. 1,1,2,2-Tetra-yl-tetramethylene tetrabromide (0.01) was added to the reaction mixture, which was refluxed for 20 hours. The mixture was filtered and the solvent was evoporated. The solid residue was recrystallized from ethyl alcohol to give compound **2c** (yield 63 %; m.p. 478–479°K). Analysis (% Calculated/found) for C_34_H_20_C_l8_O_8_ (Mw 840.1) C: 48.61/48.53, H: 2.40/2.38; IR (KBr) (ν, cm^−1^), 3067 (Ar-CH), 2972 (-CH), 2883–2894 (CHO), 1696 (C=O); ^1^H-NMR (DMSO-d_6_) δ (ppm) 5.07 (s, 8H, O-CH_2_), 7.63–7.64 (d, 4H, Ar-H), 7.98–7.99 (d, 4H, Ar-H), 10.05 (s, 4H, -CHO); 13C-NMR (DMSO-d_6_) δ (ppm) 55.99 (O-CH_2_), Ar-C: [127.3157 (C), 128.67 (C), 129.61 (C), 131.05 (CH), 135.21 (CH), 154.92 (C)], 135.05 (C=C), 188.17 (C=O); MS: m/z 882.50 (M+Na+H_2_O)^+1^.

### 2.5. Synthesis of 2,2′-(2-(1,3-bis(2-bromo-4-chloro-6-formylphenoxy)propan-2-ylidene)propane-1,3-diyl)bis (oxy)bis(3-chloro-5-bromo benzaldehyde) (***2d***)

3-Chloro-5-bromosalicylaldehyde (0.04 mol) and potassium hydroxide (0.04 mol) were refluxed in absolute ethanol for 2 hours. 1,1,2,2-Tetra-yl-tetramethylene tetrabromide (0.01) was added to the reaction mixture, which was refluxed for 20 hours. The mixture was filtered and the solvent was evaporated. The solid residue was recrystallized from DMF-ethyl alcohol (1:1) to give compound **2d** (yield 54 %; m.p. 485–486 °K). Analysis (% Calculated/found) for C_34_H_20_Br_4_Cl_4_O_8_ (Mw 1017.9) C: 40.12/40.17, H: 1.98/2.01; IR (KBr) (ν, cm^−1^), 3075 (Ar-CH), 2977 (-CH), 2796–2884 (CHO), 1698 (C=O); ^1^H-NMR (DMSO-d_6_) δ (ppm) 5.01 (s, 8H, O-CH_2_), 7.44 (s, 4H, Ar-H), 7.83 (s, 4H, Ar-H), 10.18 (s, 4H, -CHO); ^13^C-NMR (DMSO-d_6_) δ (ppm) 55.93 (O-CH_2_), Ar-C: [117.93 (C), 126.82 (C), 129.06 (C), 132.67 (CH), 135.63 (CH), 152.12 (C)], 135.45 (C=C), 188.26 (C=O).

### 2.6. Crystallographic structure determination compound ***2a***

A summary of the key crystallographic information is given in Table 1. The data was collected on a smart [15] CCD diffractomer using graphite-monochromated Mo Kα radiation at room temperature. The collected data were reduced by using the program SAINT [15] and empirical absorption correction was carried out by using the SADABS [16] program. The structure was solved by direct methods [17] as implemented in the SHELXTL system of computer programmes and refined to convergence by full matrix least-squares methods. H atoms were located geometrically and then refined isotropically with fixed displacement parameters. Atomic scattering factors used were those from the International Table for x-ray crystallography [18]. The crystal structure has been deposited at the Cambridge Crystallographic Data Center with the deposition number CCDC 686161.

## 3. Results and Discussion

**Scheme 1.**
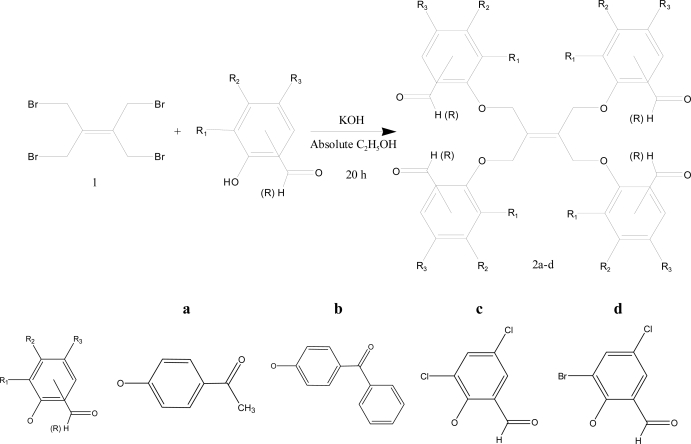
Synthesis and structures of compounds **2a–d**

The reaction of ethene-1,1,2,2-tetra-yl-tetramethylene tetra- bromide (**1**) in absolute ethanol media with the corresponding potassium salts of phenolic ketones and aldehydes (obtained by potassium hydroxide solution) gave the corresponding tetraketones **2a,b** or tetraaldehydes **2c,d** in a good yield (Scheme 1). The substitution reactions were highly selective for the tetrasubstituted products **2**, as independent of the molar ratios of ketophenol or aldehydophenol. Mono-, di- or trisubstituted products could not be obtained in this reaction. In the IR spectra of compounds **2a–d**, one sharp absorption band was seen at 1652–1698 cm^−1^ which is assigned to the carbonyl functions. The CHO Fermi doublet stretching frequency was observed at 2796–2894 cm^−1^ in the IR spectra of compounds **2c,d.**

In the ^1^H-NMR spectra of compounds **2a–d** the signals of the (-O-CH_2_) methylene groups integrating for eight protons were seen between 4.99–5.07 ppm. Aldehyde protons (CHO) of compounds **2c,d** were observed around 10.05–10.18 ppm, integrating for four protons. In the ^13^C-NMR of compounds 2a–d, OCH_2_ group was observed at 56–65 ppm. In addition, C=O and C=C functions of compounds **2a–d** appeared at 188–196 ppm and 134–135 ppm, respectively. ^1^H-NMR and ^13^C-NMR spectral data of compounds **2a–d** are presented in the Experimental section in this study.

The molecule of the title compound **2a** has a centre of symmetry located at the mid-point of the C=C double bond. The centre of symmetry C=C bond length [1.332(6) Å] agrees with the values reported in the literature [1.335(5) Å in C_50_H_36_O_8_ [19], 1.316(7) Å in C_12_H_12_N_4_S_2_ [20] and 1.318(6) Å in C_12_H_18_N_2_S_4_ [21]]. The molecular conformation is essentially described by torsion angles about the C10-C9 and C10-C11 bonds. The C10-C11-O3-C12 and C10-C9-O2-C8 torsion angles are −173.0(2)° and 159.8(2)°, respectively. The acetyl group is almost complanar with the benzene ring [C15-C17-C18=O4=179.1(3)°]. The O-C bond lengths are within normal ranges. Atoms C3 and C17, carrying the acetyl substituents, are trigonal, the sum of the three bond angles around them being 359.9(3)°. As expected, the benzene rings systems are planar, with the largest deviations being 0.0159(3) Å for C8 and 0.0132(1) Å for C15. The dihedral angle between the planes of rings are 76.43(8)°. Atom H9A of the methylene group (C9) forms an intermolecular hydrogen bond with the acetyl group O atom (O4) of a symmetry-related molecule [C9...O4^(i)^=3.159 Å; symmetry code: (i) x-1,+y,+z].

## 4. Conclusions

**Figure 1.**
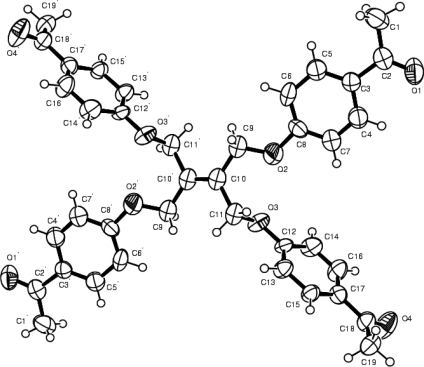
ORTEP drawing of the crystal structure of C_38_H_36_O_8_

In order to investigate the influence of the flexibility of the ligand molecule, four new carbonyl compounds have been synthesized and the crystal structure of compound **2a** was determined. Alkenetetrayltetra oxyphenylaldehyde and tetraketones were obtained by reaction four different hydroxyketones or hydroxyaldehydes and ethene-1,1,2,2-tetra-yl-tetramethylene tetra- bromide (**1**). So, we successfully prepared C=O functionalized chelates having ether bridges as a key intermediate for the synthesis of novel macromolecules containing a donor group. The compounds were prepared and identified by elemental analysis, IR, ^1^H-NMR, ^13^C-NMR and Mass spectroscopy. In addition, the crystal structure of the compound **2a**, C_38_H_36_O_8_, was determined by single crystal X-ray diffraction technique, figure 1.

## Figures and Tables

**2a:**
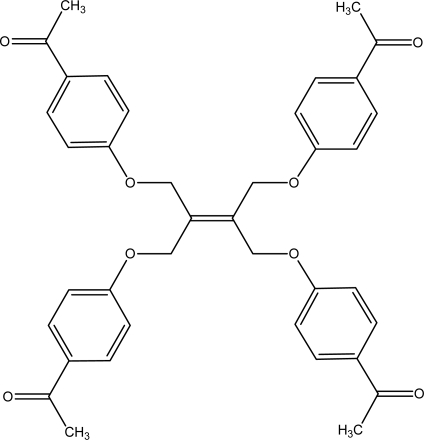


**Table 1.** Crystallographic data for C_38_H_36_O_8_

Chemical formula	C_38_H_36_O_8_	α(°)	90
Formula weight	620.67	β(°)	95.875(3)
Crystal colour, habit	Colourless, prism	γ(°)	90
Crystal system	Monoclinic	V(Å^3^)	1566.8(4)
Crystal dimensions	0.30×021×0.21	Z	2
Space group	P 21/n	D_calc_(g/cm^3^)	1.316
a(Å)	9.0395(12)	μ(Mo Kα), cm^−1^	0.092
b(Å)	12.6114(17) 13.8166(18)	No unique reflections	8396
c(Å)		No of observations	2430
		R	0.0481
		R_w_	0.0722

R = ΣllFol-|Fcll/Σ|Fol R_w_ = [(Σw(lFol-lFcl)^2^/ΣwFo^2^)]½

**Table 2.** Selected geometric parameters (Å, °) for [C_38_H_36_O_8_]

C10-C10’ 1.332(6) C15-C17-C16 118.7(3)
O1-C2 1.218(3) C15-C17-C18 123.7(3)
O4-C18 1.226(3) C16-C17-C18 117.5(3)
C4-C3-C5 118.0(3) C8-O2-C9-C10 159.8(2)
C4-C3-C2 119.9(3) C12-O3-C11-C10 −173.0(2)
C5-C3-C2 122.0(3)
